# SLEEP–WAKE: A Comprehensive Mnemonic for Systematic Screening of Sleep‐Related Issues

**DOI:** 10.1002/pdi3.70010

**Published:** 2025-06-14

**Authors:** Karen Spruyt

**Affiliations:** ^1^ INSERM Université Paris Cité Paris France

**Keywords:** assessment, child, pediatric sleep, sleep screener, tool

## 1 Introduction

Pediatric sleep disturbances have become an increasingly recognized concern due to their profound impact on a child's development, health, and well‐being [1, 2, 3, 4]. Sleep problems in children can lead to a range of consequences, including impaired cognitive function [5, 6], emotional dysregulation [7], and behavioral difficulties [8]. These challenges are particularly pronounced in children with complex needs, such as those with multiple disabilities or intellectual disability, where sleep disturbances are often pervasive and multifactorial [9, 10, 11]. Given the critical importance of sleep for development and quality of life, routine screening for sleep‐related issues in pediatric populations—especially those with developmental disabilities—is essential. The SLEEP–WAKE mnemonic offers a comprehensive framework for screening pediatric sleep problems, providing healthcare providers with an organized approach to assess the various factors contributing to sleep complaints.

## 2 The SLEEP–WAKE Prompts for Screening Pediatric Sleep Issues

The SLEEP–WAKE mnemonic encompasses key domains that contribute to the understanding of sleep patterns and disturbances, particularly in children with developmental disabilities. By focusing on these areas, healthcare providers can gather crucial information to make informed decisions about diagnosis and intervention (Figure 1).

FIGURE 1SLEEP–WAKE mnemonic with prompts.

**Figure:**
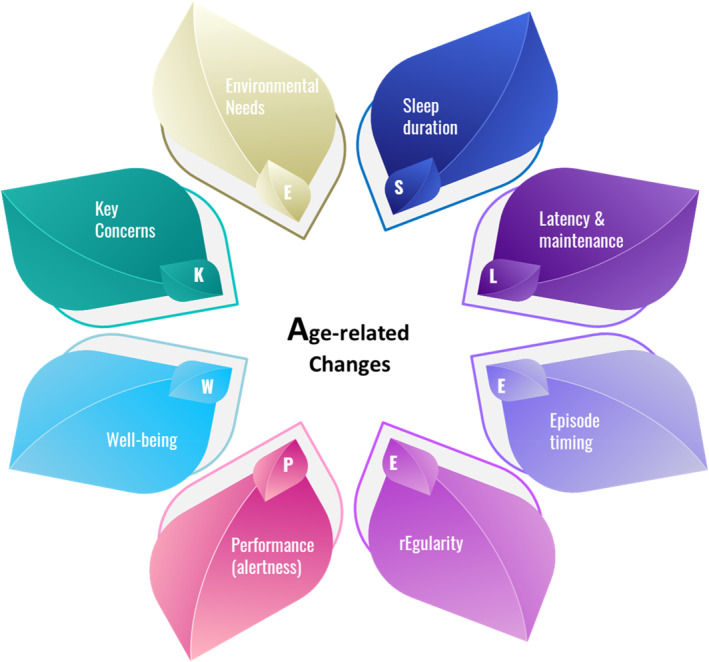

**Figure:**
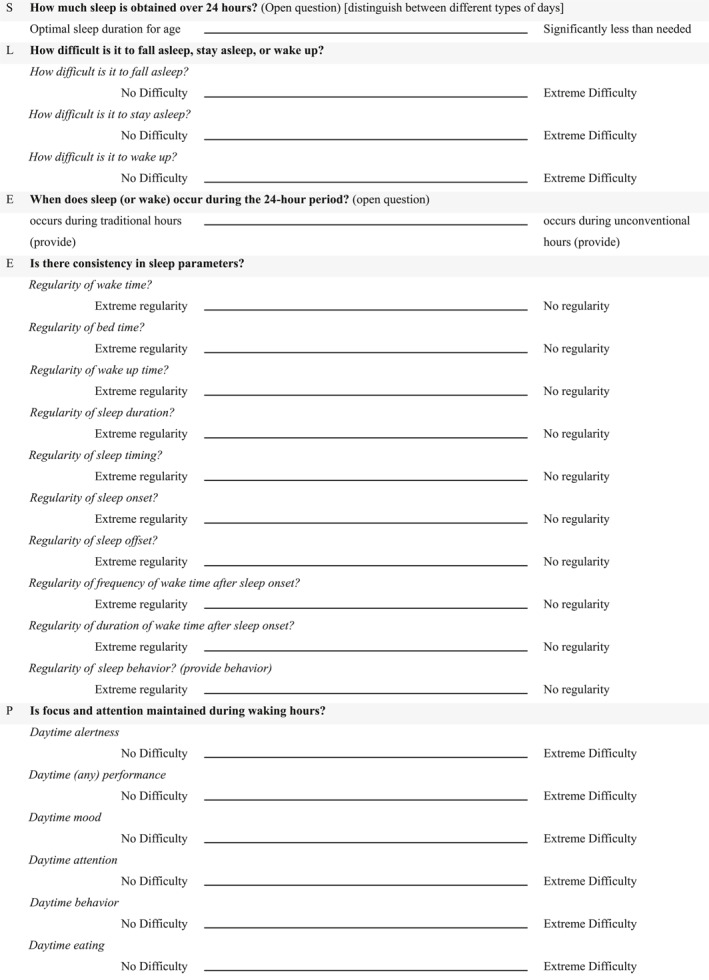

**Figure:**
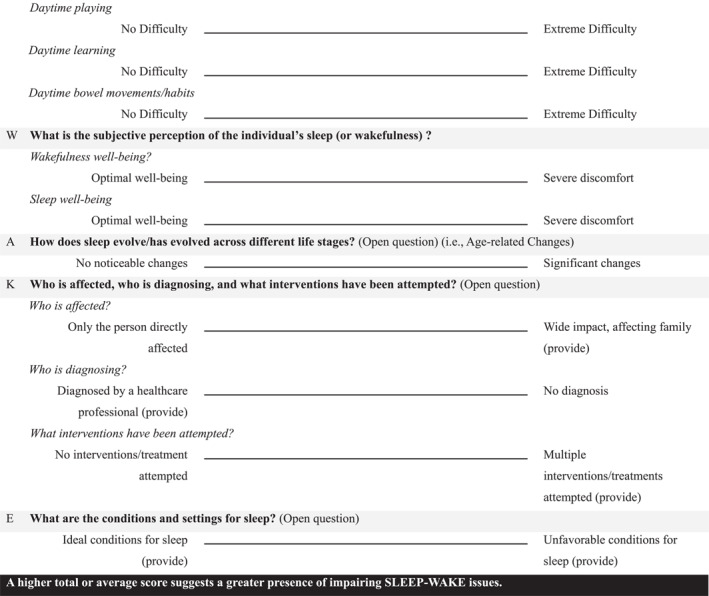

### 2.1 S: Sleep Duration

The first component, sleep duration, assesses how much sleep a child obtains over a 24‐h period [12, 13, 14]. Sleep duration is a fundamental component of sleep health, essential for understanding its significance within the 24‐h daily cycle. For children, adequate sleep duration varies by age, with younger children requiring more sleep than older children and adolescents. Children with developmental disabilities often experience fragmented or reduced sleep, which can exacerbate developmental delays, attention deficits, and emotional dysregulation, further complicating sleep habits. Therefore, understanding the child's sleep duration is crucial in identifying potential sleep disorders, such as insomnia [15] or sleep apnea [16], that may be contributing to their sleep difficulties.

In clinical practice, sleep duration can be assessed through caregiver reports, sleep diaries, or objective measures like actigraphy or trackers. A thorough assessment (i.e., 24‐h for minimal 14 days) helps determine whether the child is getting enough sleep for their developmental stage and identifies any patterns that may indicate underlying issues. When assessing sleep duration, it is important to distinguish between different types of days, as routines and behaviors often vary (Table 1).

**TABLE 1:** The SLEEP–WAKE prompts.

The prompt		Operationalization
S	Sleep duration	How much sleep is obtained over 24 h? (Open question) This can be enhanced by incorporating a 10 cm visual analog scale, which may include potential anchors: Optimal sleep duration for age versus significantly less than needed
	School day	Typically weekdays when children attend school, requiring structured schedules with earlier bedtimes and wake times.
	Weekdays (Sunday–Thursday)	General workweek days, which may include school days but can also involve variations depending on specific cultural or local customs.
	Weekend days (Friday–Saturday)	Days typically associated with more relaxed schedules, later bedtimes, and extended sleep durations.
	Holidays/vacation	Periods free from school or work obligations, often characterized by (ir)regular sleep schedules and altered routines
L	Latency and maintenance	How difficult is it to fall asleep, stay asleep, or wake up? For each, a visual analog scale (10 cm) with the potential anchors
	How difficult is it to fall asleep?	No difficulty versus extreme difficulty
	How difficult is it to stay asleep?	No difficulty versus extreme difficulty
	How difficult is it to wake up?	No difficulty versus extreme difficulty
E	Episode timing	When does sleep (or wake) occur during the 24‐h period? (Open question) This can be enhanced by incorporating a 10 cm visual analog scale, which may include potential anchors: occurs during traditional hours (provide) versus occurs during unconventional hours (provide)
E	rEgularity	Is there consistency in sleep parameters? For each sleep parameter, a visual analog scale (10 cm) with potential anchors
	Regularity of wake time?	Extreme regularity versus no regularity
	Regularity of bedtime?	Extreme regularity versus no regularity
	Regularity of wake‐up time?	Extreme regularity versus no regularity
	Regularity of sleep duration?	Extreme regularity versus no regularity
	Regularity of sleep timing?	Extreme regularity versus no regularity
	Regularity of sleep onset?	Extreme regularity versus no regularity
	Regularity of sleep offset?	Extreme regularity versus no regularity
	Regularity of frequency of wake time after sleep onset?	Extreme regularity versus no regularity
	Regularity of duration of wake time after sleep onset?	Extreme regularity versus no regularity
	Regularity of sleep behavior?	Extreme regularity versus no regularity
*P*	Performance (alertness)	Is focus and attention maintained during waking hours? For each wake parameter, a visual analog scale (10 cm) with potential anchors
	Daytime alertness	No difficulty versus extreme difficulty
	Daytime performance	No difficulty versus extreme difficulty
	Daytime mood	No difficulty versus extreme difficulty
	Daytime attention	No difficulty versus extreme difficulty
	Daytime behavior	No difficulty versus extreme difficulty
	Daytime eating	No difficulty versus extreme difficulty
	Daytime playing	No difficulty versus extreme difficulty
	Daytime learning	No difficulty versus extreme difficulty
	Daytime bowel movements/habits	No difficulty versus extreme difficulty
W	Well‐being	What is the subjective perception of the individual's sleep (or wakefulness) ? For each, a visual analog scale (10 cm) with potential anchors
	Wakefulness well‐being?	Optimal well‐being versus severe discomfort
	Sleep well‐being?	Optimal well‐being versus severe discomfort
A	Age‐related changes	How does sleep evolve/has evolved across different life stages? (Open question) This can be enhanced by incorporating a 10 cm visual analog scale, which may include potential anchors: No noticeable changes versus significant changes
K	Key concerns	Who is affected, who is diagnosing, and what interventions have been attempted? (Open question) This can be enhanced by incorporating a 10 cm visual analog scale, which may include potential anchors: Only the person directly affected versus wide impact, affecting family (provide); diagnosed by a healthcare professional (provide) versus No diagnosis; No interventions/treatment attempted versus multiple interventions/treatments attempted (provide)
E	Environmental needs	What are the conditions and settings for sleep? (Open question) This can be enhanced by incorporating a 10 cm visual analog scale, which may include potential anchors: Ideal conditions for sleep (provide) versus unfavorable conditions for sleep (provide)

*Note:* A higher total or average score suggests a greater presence of impairing SLEEP‐WAKE issues.

### 2.2 L: Latency and Maintenance

The latency and maintenance of sleep refer to how difficult it is for a child to fall asleep, stay asleep, and maintain sleep throughout the night or during nap times. Sleep latency is the time it takes for a child to transition from wakefulness to sleep, while maintenance refers to the ability to stay asleep once sleep onset occurs. Children with developmental disabilities often struggle with both sleep onset and sleep maintenance, due to factors such as anxiety, physical discomfort, or neurological conditions, among others [10]. Disruptions in sleep maintenance can lead to frequent nighttime awakenings and concerning nighttime behaviors, resulting in fragmented sleep and excessive daytime sleepiness [11].

Screening for sleep latency and maintenance issues can be conducted using caregiver observations, clinical interviews, and sleep questionnaires [17, 18]. Additionally, tools such as sleep trackers and videosomnography can provide valuable objective data to complement these assessments. The information gathered can help identify potential causes of sleep disturbances, such as pain, sensory processing issues, or co‐occurring medical conditions like epilepsy [19].

### 2.3 E: Episode Timing

The episode timing component focuses on when sleep occurs during the 24‐h period. It is essential to understand not only how long a child sleeps but also when they sleep. Children with developmental disabilities may experience atypical sleep habits, such as irregular sleep‐wake cycles or reversed circadian rhythms [10, 11]. These disturbances can be exacerbated by medical treatments, environmental factors, or the child's unique neurological, sensory, or nutritional/gut needs. In some cases, it may also be prudent to assess the timing of wake episodes for a comprehensive evaluation.

Assessing the timing of sleep episodes provides valuable insights into potential disruptions in circadian rhythms, which can be linked to conditions such as delayed sleep phase disorder or the impact of environmental factors like light exposure. Healthcare providers can use sleep logs, diaries, and digital tools like wearable devices to track sleep timing and identify patterns that may need intervention.

### 2.4 E: rEgularity

Regularity refers to the consistency of the sleep state, including sleep and wake times, sleep duration, and other sleep parameters. A consistent sleep state is essential for maintaining healthy sleep and supporting optimal circadian rhythms [20]. In children with developmental disabilities, for instance, irregular sleep patterns are common, with varying sleep onset times, fluctuating durations, and inconsistent wake‐up times [10, 11]. These irregularities can have detrimental effects on mood, cognition, and behavior, further complicating health, care and development [21, 22].

Regularity can be assessed by monitoring sleep over an extended period, either through caregiver reports or by using objective measures such as actigraphy or trackers. Regularity is often a key focus of intervention; for example, establishing a consistent sleep‐wake state can improve both sleep quality and daytime functioning.

### 2.5 P: Performance (Alertness)

Performance, or alertness, is an important indicator of how well a child is functioning during waking hours. Sleep disturbances can lead to daytime sleepiness, reduced attention span, irritability and difficulty focusing, which are particularly concerning in children with developmental disabilities, who may already experience challenges in these areas. The impact of sleep issues on a child's performance is not only related to cognitive abilities [5, 23] but also emotional and behavioral regulation [24].

Assessment of performance involves evaluating the child's level of alertness and attention during the day. This can be done through caregiver reports, school evaluations, medication assessments, and behavioral assessments. In children with significant developmental delays or neurological impairments, it is important to consider the role that sleep may play in exacerbating these challenges.

### 2.6 W: Well‐Being

Well‐being refers to the subjective perception of an individual's sleep (or wakefulness) state. This domain acknowledges that sleep issues can affect not only the child's health/performance but also their overall satisfaction and quality of life [25]. Children with developmental disabilities often struggle to express their discomfort or sleep‐related issues verbally, making it crucial to assess caregivers' perceptions of the children's sleep well‐being. For example, caregivers can provide insight into the child's willingness to continue sleeping or their tolerance for subpar sleep conditions.

Caregivers' perspectives on their children's sleep are invaluable, as they provide a broader view of the child's overall health and functioning. When the child reaches an appropriate age, they can be encouraged to share their experiences. Questionnaires and interviews with caregiver (or child) can help identify whether the child's sleep issues are causing distress, frustration, or other emotional difficulties that need to be addressed.

### 2.7 A: Age‐Related Changes

Age‐related changes recognize that sleep evolves as children grow [12, 13, 14]. Sleep needs and characteristics change across different life stages, and this is particularly relevant in children with developmental disabilities, who may have unique developmental trajectories. Understanding how sleep has evolved for a particular child over time is essential for identifying when deviations from “typical” sleep states occur.

For example, children with complex needs may experience delayed sleep onset or irregular sleep patterns at an earlier age compared to neurotypical peers. Tracking these changes over time can help healthcare providers identify sleep disorders and intervene early, promoting better sleep health throughout development. Age‐related changes, particularly those that occur during puberty, can be effectively managed to prevent them from developing into pathological or abnormal conditions.

### 2.8 K: Key Concerns

Key concerns focus on understanding who is affected, who is diagnosing the issue, and what interventions have been attempted. Pediatric sleep issues often involve a diverse group of stakeholders, including the child, their family, parents, caregivers, healthcare providers, and specialists. Identifying the key concerns of each individual involved in the child's care is crucial for developing a comprehensive collaborative approach to treatment.

It is important to consider both the child's medical, treatment history and the challenges faced by caregivers in managing sleep issues. Healthcare providers should work closely with families to understand their concerns and ensure that interventions are appropriate and effective for the specific needs of the child and family.

### 2.9 E: Environmental Needs

Finally, Environmental needs assess the physical and sensory environment in which the child sleeps. Factors such as noise, light exposure, room/body temperature, and sleep surface can significantly impact sleep quality [26, 27], particularly for children with developmental issues who may have heightened sensitivities. Evaluating the sleep setting allows healthcare providers to recommend modifications that can improve sleep conditions and enhance the child's overall sleep experience [28].

Interventions may include adjustments to the sleep environment, such as reducing light exposure before bedtime, using weighted blankets, or creating a calming nighttime routine. These environmental changes can help children (with complex needs) achieve better sleep, promoting improved health and development.

## 3 Conclusion

The SLEEP‐WAKE prompts (Figure 1) provides a comprehensive, structured approach to screening pediatric sleep issues, particularly in children with developmental disabilities. By addressing prompts such as sleep duration, latency, timing, regularity, performance, well‐being, age‐related changes, key concerns, and environmental needs, healthcare providers can gain a holistic understanding of a child's sleep health. Routine screening using this framework helps identify sleep issues early, ensuring timely intervention and improved outcomes. Given the profound impact of sleep on a child's physical, cognitive, and emotional well‐being, incorporating the SLEEP‐WAKE approach into routine pediatric care is essential for optimizing sleep health in children (with complex needs). It facilitates the development of culturally sensitive sleep interventions and guidelines tailored to diverse populations, ensuring equitable care for all children.

## Author Contributions

The author takes full responsibility for this article.

## Ethics Statement

The author has nothing to report.

## Conflicts of Interest

Prof. Karen Spruyt is deputy editor‐in‐chief of *Pediatric Discovery*. And she was excluded from all editorial decision‐making related to the acceptance for publication. Apart from this, she declares no conflict of interest.

## Data Availability

The author has nothing to report.
